# Are NCAM deficient mice an animal model for schizophrenia?

**DOI:** 10.3389/fnbeh.2012.00043

**Published:** 2012-07-17

**Authors:** Anne Albrecht, Oliver Stork

**Affiliations:** ^1^Department of Genetics and Molecular Neurobiology, Institute of Biology, Otto-von-Guericke University MagdeburgMagdeburg, Germany; ^2^Center for Behavioral Brain ScienceMagdeburg, Germany

**Keywords:** NCAM, nest building, social interaction, latent inhibition, schizophrenia

## Abstract

Genetic and biomarker studies in patients have identified the Neural Cell Adhesion Molecule (NCAM) and its associated polysialic acid (PSA) as a susceptibility factors for schizophrenia. NCAM and polysialtransferase mutant mice have been generated that may serve as animal models for this disorder and allow to investigate underlying neurodevelopmental alterations. Indeed, various schizophrenia-relevant morphological, cognitive and emotional deficits have been observed in these mutants. Here we studied social interaction and attention of NCAM null mutant (NCAM^−/−^) mice as further hallmarks of schizophrenia. Nest building, which is generally associated with social behavior in rodents, was severely impaired, as NCAM^−/−^ mice continuously collected smaller amounts of nest building material than their wild type littermates and built nests of poorer quality. However, social approach tested in a three—compartment—box was not affected and latent inhibition of Pavlovian fear memory was not disturbed in NCAM^−/−^ mice. Although NCAM deficient mice do not display a typical schizophrenia-like phenotype, they may be useful for studying specific endophenotypes with relevance to the disease.

## Introduction

Genetic linkage and association studies suggest a polygenetic contribution of multiple risk genes that influence the susceptibility to develop schizophrenia (Gejman et al., 2011). Candidates comprise genes involved in synaptic plasticity, signal transduction, neurite outgrowth, and cell adhesion (Robertson et al., 2006). Single gene mutations are thought to contribute to less then 0.1% to the heritability of schizophrenia. Still, mice with genetic manipulations of identified susceptibility genes may provide valuable tools for better understanding the neurobiology of schizophrenia and the development of new therapeutic strategies. Testing of endophenotypes related to schizophrenia in animal models generally is focused on deficits in cognition, attention, and negative symptoms like emotional blunting or social dysfunction (Kellendonk et al., 2009; Mazzongini et al., 2009; Amann et al., 2010). The analysis of morphological changes in such rodent models has provided strong support for the neurodevelopmental hypothesis of schizophrenia (Robertson et al., 2006; Jaaro-Peled et al., 2010; Lu et al., 2011).

One molecule widely involved in neural development is the Neural Cell Adhesion Molecule (NCAM), which mediates Ca^2+^-independent cell-cell and cell-extracellular matrix interactions during proliferation, cell migration, neurite outgrowth, axon fasciculation, and synaptic remodeling (Fields and Itoh, 1996; Ronn et al., 2000; Povlsen and Ditlevsen, 2010). Altered concentrations of NCAM isoforms and soluble NCAM fragments have been frequently observed in the cerebrospinal fluid of schizophrenic patients (Vawter, 2000; Brennaman and Maness, 2010), as well as in hippocampus, prefrontal cortex (PFC) and other cortical areas (Vawter et al., 2001; Gibbons et al., 2009; Gray et al., 2010). Moreover, genetic association studies have identified NCAM as candidate susceptibility gene for schizophrenia, although findings were inconsistent (Vicente et al., 1997; Sullivan et al., 2007), raising the question whether NCAM may play a causal role in development of schizophrenia symptoms.

This may be addressed in mice with a targeted disruption of the NCAM gene (NCAM^−/−^ mice). These animals display moderate changes of brain morphology, reduced rhythmic network activity in the hippocampus, mild cognitive impairment and emotional changes including increased aggression, anxiety, and anti-depressant like responses (Cremer et al., 1994, 1997; Stork et al., 1997, 1999, 2000; Albrecht et al., 2010).

In this study we further investigated social behavior and attention deficits as core domains of schizophrenia, that previously could not be adequately assessed due to confounds from increased aggression (Stork et al., 1997) and memory deficits (Stork et al., 2000). Firstly, nest building behavior as a typical rodent behavioral feature linked to social activity (Crawley, 2007; Amann et al., 2010), and secondly social approach towards an unknown interaction partner in a three—chambered apparatus was assessed (Crawley, 2007), allowing to test for social behavior without interference by aggressive encounters in NCAM^−/−^ mice. Recently, we could demonstrate that deficits in both auditory cued and contextual fear conditioning can be overcome by a pre-exposure of NCAM^−/−^ mice to a neutral tone (CS-) and the training context (Albrecht et al., 2010). Engaging such pre-exposure strategies, we were now able to investigate attention processes in NCAM^−/−^ mice in a latent inhibition paradigm without adverse effects of fear memory deficits.

As NCAM^−/−^ mice displayed disturbed nest building, but normal approach towards conspecifics as well as normal latent inhibition in the current study, we could not sustain the hypothesis that loss of NCAM is a critical factor for schizophrenia-like behavioral changes in general.

## Materials and methods

NCAM mutants (backcrossed to C57Bl/6BomTac for >12 generations) from heterozygous breeding were raised in groups of 2–6 under standard laboratory conditions with a 12 h day/night cycle (lights on at 7.00 P.M. with a 30 min dawn phase) and food and water ad libitum. Genotypes were determined by polymerase chain reaction as described previously (Stork et al., 2000). Mice were separated one week before the experiments and housed individually throughout the behavioral assessment. Testing of animals was always done during the dark cycle between 10.00 A.M. and 6.00 P.M. All experiments were in accordance with the European and German regulations and approved by the Landesverwaltungsamt Sachsonia-Anhalt AZ 2–441 and 2–618.

### Social behavior

#### Nest building

Male adult NCAM^+/+^ (*N* = 14) and NCAM^−/−^ mice (*N* = 18; 10–16 weeks of age) were provided with a 2.2 g pellet of bedding cotton (Nestlets, 5 × 5 cm, EBECO, Castrop-Rauxel, Germany) accessible through the food hopper. The amount of cotton collected for nest building was determined over time. Nest quality was judged by a trained observer, who was blind for the animals' genotype, using a score system adapted from (Kalueff et al., 2006). (0 points = no nest through 4 points = hooded nest). In the same manner, nest quality was rated after paper tissue was provided directly in the cage (*N* = 6 for NCAM^−/−^; *N* = 7 for NCAM^+/+^) to control for potential differences related to the nesting material or material gathering. Additionally, body temperature was determined both with nesting material in the cage and 24 h after nest removal. A small animal thermometer (TD-300; Shibaura Electric Co. Ltd., Tokyo, Japan) pre-warmed to 37°C in baby oil was introduced rectally. Measurements were always done between 12:00 and 13:00 and completed within 2 min to avoid stress-induced hypothermia.

#### Social approach

NCAM^−/−^ (*N* = 8) and NCAM^+/+^ mice (*N* = 10) were introduced into the center compartment of a 40 × 60 cm three-compartment chamber. Transparent acryl plates with 6 × 6 cm openings allowed free access to the neighbouring compartments each containing a perforated acrylic glass cylinder (diameter: 8 cm) for housing an interaction partner. After 5 min of free exploration test mice were confined to the center compartment for 1 min while an unfamiliar male wild type mouse serving as interaction partner was placed in one of the cylinders while the cylinder in the other compartment remained empty. The test mouse was allowed to explore all compartments for 30 min during which movements were video recorded and tracked with ANY-maze (Stoelting, Wood Dale, IL, USA). An observer blind for genotype of the test mouse and location of the interaction partner scored direct contacts with key presses.

### Latent inhibition

To generate latent inhibition, NCAM^−/−^ mice (*N* = 12) and their NCAM^+/+^ littermates (*N* = 12) were pre-exposed to 40 CS+ tones (10 kHz, 85 dB SPL), which were presented in four sessions of each 10 over two days. In each session, after 2 min of habituation, ten 10 s stimuli were presented with inter-stimulus-intervals (ISI) of 20 s (CS+ PE group). A second group of NCAM^−/−^ (*N* = 12) and NCAM^+/+^ mice (*N* = 12) received pre-exposure to 40 neutral acoustic stimuli of 2.5 kHz (CS– PE group) and a third group of NCAM^−/−^ (*N* = 12) and NCAM^+/+^ mice (*N* = 12) was pre-exposed only to the training context, but no tone (Cxt PE). In all groups, fear conditioning on day three was done by pairing three CS+ (9 s, 10 kHz, 85 dB) with mild electric foot shocks (1 s, 0.4 mA; ISI 20 s). Fear memory retrieval was tested 24 h later in a neutral context. After 2 min sets of each four CS− and four CS+ with 20 s ISI were presented for auditory cued fear memory. On the next day, memory to the training context was tested for 2 min. Freezing behavior as a measure of fear memory was determined with a photo beam detection according to previously established parameters (Laxmi et al., 2003; Albrecht et al., 2010).

## Results

### Social behavior

#### Nest building

**Figure 1:**
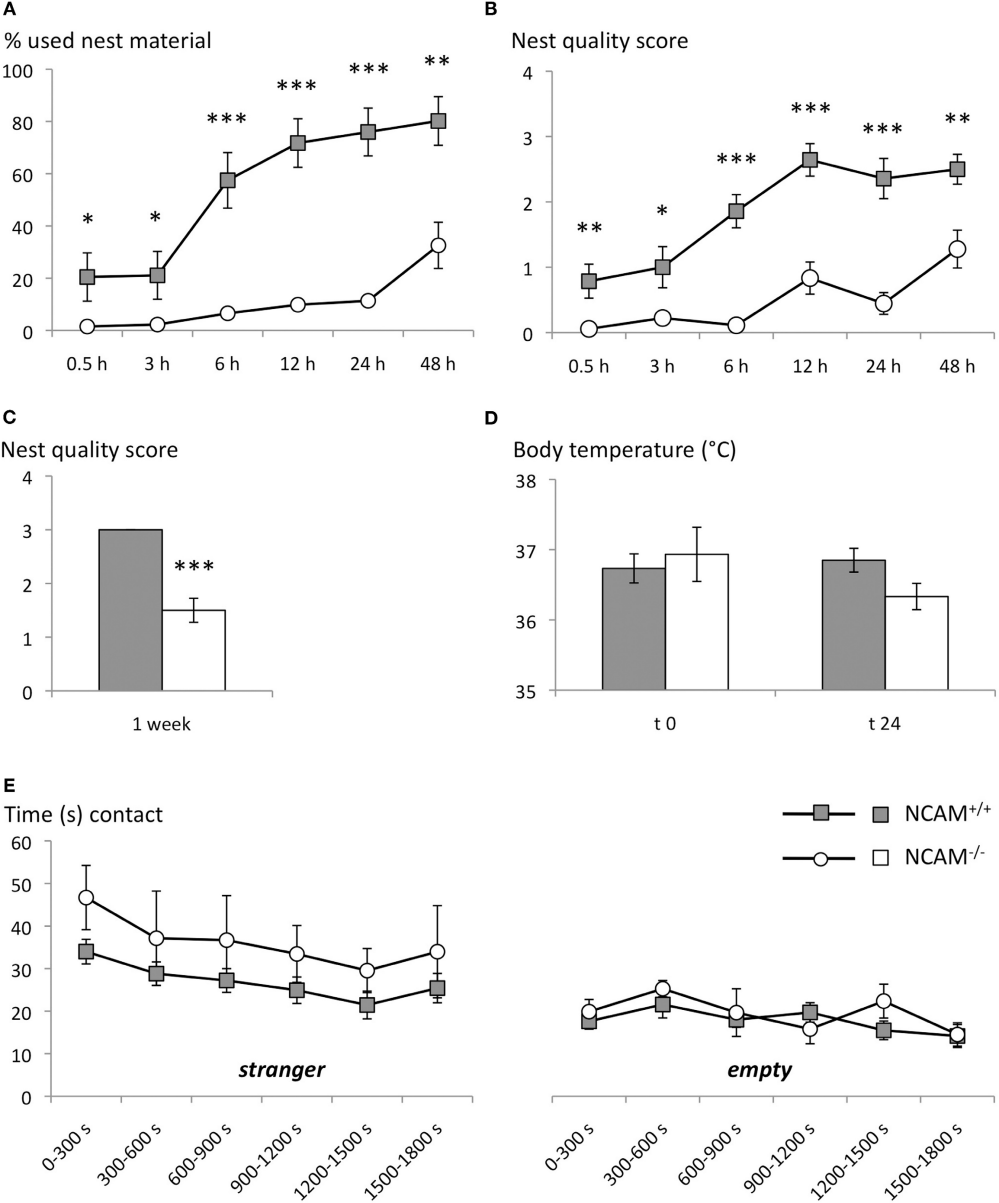
**Disturbed nest building, but normal social approach in NCAM^−/−^ mice. (A)** NCAM^−/−^ mice collected continuously less amounts of cotton tissue from the food hopper than their wild type littermates. **(B)** Consistently, NCAM^−/−^ mice built only primitive flat nests. **(C)** Similar, when tissue paper was provided as nesting material in the cage NCAM^−/−^ mice built only nests of poor quality, as assessed after one week. **(D)** The body temperature of NCAM^−/−^ mice did not differ significantly from NCAM^+/+^ mice either with a nest available at t 0 or 24 h after removal of the nesting material at t 24. **(E)** In a social interaction test NCAM^−/−^ mice displayed no deficits in direct approaches towards a tube containing the stranger mouse (left column) and showed comparable amounts of contacts towards an empty tube compared to NCAM^+/+^ mice. Values are indicated as mean ± SEM. ^*^Significant difference between genotypes with *p* < 0.05; ^**^p < 0.01; ^***^p < 0.001.

The analysis of cotton sampling activity showed a significant effect of genotype on the amount of nesting material used [ANOVA with effect of gene *F*_(1, 30)_ = 35.887; *p* < 0.001]. Over time, animals of both genotypes collected more nest material [repeated measurement ANOVA with time as factor: time *F*_(5, 150)_ = 31.895, *p* < 0.001], but a genotype effect was observed over time [time × genotype interaction *F*_(5, 150)_ = 9.928, *p* < 0.001]. In fact even after 48 h NCAM^−/−^ mice had used only 32 ± 8.8% (mean ± SEM), while their NCAM^+/+^ littermates had taken 80 ± 9.3% of the provided cotton (Figure 1A).

Moreover, NCAM^−/−^ mice built only primitive nests, while NCAM^+/+^ animals managed to form more complex, cup-shaped nests with the collected cotton [ANOVA for effect of gene: *F*_(1, 30)_ = 36.872; *p* < 0.001]. It took all animals several hours to construct their nests [repeated measurement ANOVA with time as factor: time *F*_(5, 150)_ = 25.552; *p* < 0.001], but the genotype effect was stable over time [time × genotype: *F*_(5, 150)_ = 5.004, *p* < 0.001; Figure 1B].

Similarly, when the nesting material was provided directly in the cage, NCAM^−/−^ animals built nests of poor quality, revealing a significant effect of the genotype [ANOVA for effect of gene: *F*_(1, 12)_ = 53.269; *p* < 0.001; Figure 1C].

Since an altered function of the serotonergic system has been described in NCAM mutant mice (Stork et al., 2000) that might affect nesting behavior by altering thermoregulation, we measured the body temperature in the presence of the nest (t 0) and 24 h after its removal (t 24). No significant difference between genotypes could be observed [ANOVA for effect of gene at t 0: *F*_(1, 10)_ = 0.209; *p* = 0.675; at t 24: *F*_(1, 10)_ = 4.197; *p* = 0.068], suggesting a normal thermoregulatory control in NCAM mutant mice (Figure 1D).

#### Social approach

Over the 30 min test period, repeated measures ANOVA with 5 min segments as repeated factor and genotype as inter-subject factor revealed no difference between genotypes for the time spent either in the compartment with the stranger mouse [*F*_(1, 16)_ = 1.381; *p* = 0.257] or for time spent in the empty compartment [*F*_(1, 16)_ = 2.035; *p* = 0.173]. Mice of both genotypes spent more time in direct contact with the tube containing the stranger mouse, with contact time decreasing over the test period [segment: *F*_(5, 80)_ = 3.662; *p* = 0.005; genotype: *F*_(1, 16)_ = 1.666; *p* = 0.215]. Time spent in direct contact with an empty tube also decreased over the test segments [*F*_(5, 80)_ = 2.756; *p* = 0.024], but again no difference between genotypes was detected [*F*_(1, 16)_ = 0.461; *p* = 0.507; Figure 1E].

Together, NCAM^−/−^ mice showed social affiliation behavior towards a stranger mouse comparable to their wild type littermates, although nest building was disturbed in the mutant mice.

### Latent inhibition

Latent inhibition of auditory cued fear memory was achieved in NCAM^−/−^ and NCAM^+/+^ mice by pre-exposure to the conditioned tone before training (CS + PE), and compared to control groups that received pre-exposure to the context (Cxt PE) or a neutral tone (CS – PE).

**Figure 2:**
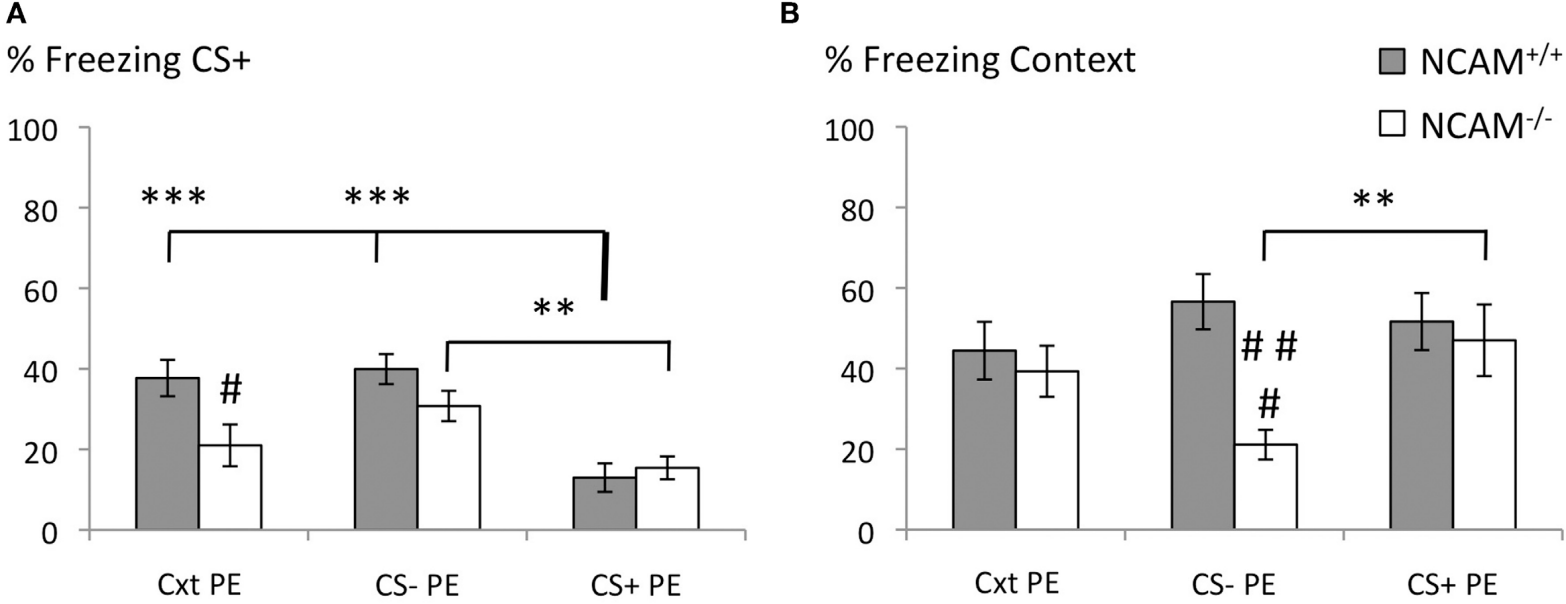
**No deficits of latent inhibition in NCAM^−/−^ mice. (A)** Freezing response to the conditioned stimulus (CS+) was reduced in both genotypes when pre-exposure to the CS+ was conducted (CS + PE). NCAM^−/−^ mice show reduced freezing when pre-exposure occurred only to the training context (Cxt PE). **(B)** Freezing response to the context was reduced in NCAM deficient mice when pre-exposed to the CS– only (CS–PE). Values are indicated as mean ± SEM. ^**^Significant difference between pre-exposure procedures, *p* < 0.01; ^***^p < 0.001. ^#^Significant difference between genotypes, *p* < 0.05; ^###^,*p* < 0.001.

Freezing to the CS+ during the retrieval was affected by genotype and pre-exposure [Two-Way-ANOVA for genotype: *F*_(1, 65)_ = 5.829; *p* = 0.019; for group: *F*_(2, 65)_ = 15.295; *p* = 0.000; genotype x group interaction: *F*_(2, 65)_ = 2.946; *p* = 0.06]. Mice of both genotypes showed comparably low levels of freezing to the CS+ after CS+ pre-exposure (mean ± SEM: 12.99 ± 3.53 for NCAM^+/+^ and 15.42 ± 2.84 for NCAM^−/−^ mice). Targeted comparison of pre-exposure group effects in each genotype demonstrated successful latent inhibition in NCAM^+/+^ mice with significantly higher levels of freezing after context pre-exposure (mean ± SEM: 37.71 ± 4.52; *p* = 0.000 to CS + PE) and CS– pre-exposure (mean ± SEM: 39.93 ± 3.73; *p* = 0.000 to CS + PE). In NCAM^−/−^ mice freezing to the CS+ was also significantly increased after CS– pre-exposure (mean ± SEM: 30.76 ± 3.77; *p* = 0.009 to CS + PE). However, after context pre-exposure, freezing to the CS+ was reduced in NCAM^−/−^ mice (mean ± SEM: 20.98 ± 5.20; n.s. to CS + PE) compared to their wild type littermates (*p* = 0.024; Figure 2A).

Freezing to the context, in which the training occurred, was not affected by the different pre-exposure paradigms [Two-Way-ANOVA; effect of group: *F*_(2, 65)_ = 1.252; *p* = 0.293]. However, ANOVA revealed an effect of genotype [*F*_(1, 65)_ = 7.242; *p* = 0.009] and a genotype × group interaction [*F*_(2, 65)_ = 3.336, *p* = 0.042] on freezing towards the conditioned context. While NCAM^+/+^ mice showed comparable amounts of freezing in each pre-exposure group (mean ± SEM: 44.44 ± 7.16 for context pre-exposure; mean ± SEM: 56.60 ± 6.87 for CS– pre-exposure; mean ± SEM: 51.67 ± 7.08 for CS+ pre-exposure), they displayed reduced freezing to the context when pre-exposed to the CS– (mean ± SEM: 21.11 ± 3.69 for CS– pre-exposure vs. 47.01 ± 8.89 for CS+ pre-exposure; *p* = 0.009; *p* = 0.000 NCAM^+/+^ vs. NCAM^−/−^ in CS+ pre-exposure; Figure 2B).

Together, these results demonstrate that in NCAM^−/−^ mice normal latent inhibition of auditory cued fear memory is possible, in spite of specific memory deficits to both CS+ and the training context.

## Discussion

In the current study we tested the hypothesis that previously observed neurodevelopmental, cognitive and emotional deficits in NCAM^−/−^ mice may be part of a complex phenotype of schizophrenic-like behavior. To this end, we further analyzed social interaction and attention that previously could not be adequately assessed due to confounds from increased aggression (Stork et al., 1997) and memory deficits (Stork et al., 2000). Our results reveal disturbed nest building, but normal approach towards conspecifics in NCAM^−/−^ mice, as well as normal latent inhibition compared to wild types and to mutants with attenuated memory deficits. Hence although several behavioral domains affected in schizophrenia are sensitive to NCAM mutation, the overall phenotypic profile of NCAM^−/−^ mice does not fully resemble a schizophrenic-like pattern.

A genetic mouse model cannot be expected to reproduce all aspects of schizophrenia, especially considering the variability among schizophrenia patients and similarities to other disorders. For example, impaired social interaction is also a core feature of autism spectrum disorders and there exists an overlap between autistic and, especially, negative symptoms of schizophrenia (e.g., affective flattening, social impairments, and poor communication) as well as a shared difficulty to adapt to novel and stressful situations (see Tordjman et al., 2007 for review). In fact, several well established models display morphological and behavioral alterations in some, but not all domains (e.g., disrupted in schizophrenia-1 (DISC-1), neuregulin-1 (NRG-1), NMDA receptor subunit 1 (NR1) or catechol-o-methyltransferase (COMT); (for review, see: Gainetdinov et al., 2001; O'Tuathaigh et al., 2007; Kellendonk et al., 2009; Jaaro-Peled et al., 2010; Lu et al., 2011). Nevertheless, some of the behavioral and morphological phenotypes associated with schizophrenia have been observed in NCAM^−/−^ mice (see Table 1 for overview). These include morphological alterations like a reduced olfactory bulb size (Cremer et al., 1994; Stork et al., 2000) and disturbed mossy fiber architecture (Cremer et al., 1997), which have likewise been reported in schizophrenic patients (Turetsky et al., 2000; Kolomeets et al., 2007; Tamminga et al., 2010). Alterations in neural connectivity are also related to changes in oscillatory activity in the theta and gamma frequency range that are frequently observed in schizophrenic patients (Uhlhaas et al., 2008; Lisman, 2011). In NCAM^−/−^ mice, a reduced amygdalo-hippocampal theta synchronization is observed during fear memory retrieval (Albrecht et al., 2010).

**Table 1:** **Phenotypes of NCAM^−/−^ mice relevant to schizophrenia**.

**Functional domain**	**Factor tested**	**Findings in NCAM^−/−^ mice**	**Reference**
**BRAIN MORPHOLOGY**			
	Olfactory bulb Hippocampus	reduced size, disturbed migration from SVZ	Cremer et al., 1994; Stork et al., 2000
		disturbed mossy fiber architecture	Cremer et al., 1994, 1997
**ELECTROPHYSIOLOGY**			
	Theta oscillation	reduced hippocampal theta synchronization	Albrecht et al., 2010
**BEHAVIOR**			
Locomotion	Open field activity	↑	Stork et al., 2000
Sensory gating	PPI	=	Plappert et al., 2005
Attention	Latent inhibition	=	Figure 2
Cognitive functions	Morris Water Maze	↓ ↓^*^ ↓↓ by stress^*^	Cremer et al., 1994; Bisaz et al., 2011; Bisaz and Sandi, 2012
	Contextual fear conditioning	↓ / = ↓↓ by stress	Albrecht et al., 2010; Stork et al., 2000
	Cued fear conditioning	↓ / = ↓^*^	Albrecht et al., 2010; Stork et al., 2000; Bisaz and Sandi, 2010
Social interaction	Nest building	↓	Figures 1A–D
	3-compartment test	=	Figure 1E
**NEUROTRANSMITTER SIGNALING**			
Serotonergic signaling	5HT1A receptor expression	=	Stork et al., 1999
	Sensitivity to serotonergic compounds	↑	Stork et al., 2000
Dopaminergic signaling	D2 receptor expression	↑	Xiao et al., 2009
	Sensitivity to dopaminergic compounds	↑	Xiao et al., 2009

Comparison of morphological (Turetsky et al., 2000; Kolomeets et al., 2007; Tamminga et al., 2010) and electrophysiological alterations (Uhlhaas et al., 2008; Lisman, 2011) found in schizophrenic patients as well as behavioral assays relevant for schizophrenia- associated symptoms (Mazzongini et al., 2009; Amann et al., 2010) and altered neurotransmitter signaling (Joober et al., 2002) with alterations in NCAM^−/−^ mice described previously and in this study. ^*^Indicates findings from conditional, forebrain-specific NCAM deficient mice. Some cognitive deficits in constitutive and conditional NCAM^−/−^ mice can be further enhanced by stress.

NCAM^−/−^ mice also display some behavioral changes potentially related to schizophrenia, e.g., hyperactivity and impaired spatial learning (Cremer et al., 1994; Stork et al., 2000). Interestingly, cognitive deficits in contextual fear conditioning were augmented in NCAM^−/−^ mice using more stressful paradigms (Albrecht et al., 2010). In conditional NCAM deficient mice with a forebrain-specific postnatal reduction of NCAM expression disturbed cognitive functions are also evident in fear conditioning as well as spatial and reversal learning in the Morris water maze (Bisaz and Sandi, 2010; Bisaz et al., 2011). This suggests that disturbance of acute NCAM-mediated cell recognition events rather than developmental effects of NCAM deficiency may cause these cognitive impairments. Moreover, subchronic stress further enhances deficits in water maze reversal learning in the conditional NCAM deficient mice but has no effect on their wildtype littermates (Bisaz and Sandi, 2012), suggesting that reduced NCAM expression may serve as a vulnerability factor for expression of behavioral impairments after stressful experience. This is in line with observations in schizophrenia, where psychosocial stress can trigger the initial expression or exacerbation of symptoms (Walker et al., 2008).

However, the changes reported so far are not disease-specific and alterations in behavioral domains not directly relevant for schizophrenia could compromise the analysis in these animals. In the current study we employed paradigms in which confounding conditions are minimized to analyze two core symptoms of schizophrenia, loss of sociability and attention deficits.

Considering that NCAM^−/−^ mice show increased aggressive behavior in an intruder aggression paradigm, we engaged non-confrontative paradigms to test social behavior without aggressive encounters. Firstly, we assessed nest building behavior as a typical rodent behavioral feature linked to social activity (Crawley, 2007; Amann et al., 2010). Nest building behavior is a species-specific innate behavioral response important for thermoregulatory control and critical for the fitness of wild and laboratory mice (DeLuca et al., 1989; Bult and Lynch, 1997). However, we observed in NCAM^−/−^ mice a severe quantitative and qualitative disturbance of nest building that was independent of any change in body temperature or response to novelty. Nest building is disturbed in various mutants with social interaction deficits (Moretti et al., 2005; Crawley, 2007; Samaco et al., 2008; Etherton et al., 2009) and also observed in other mouse models for schizophrenia (Belforte et al., 2010). Therefore secondly, we analyzed social approach towards an unknown interaction partner in a three—chambered apparatus, which allows for sensory contact but prevents aggressive encounters (Crawley, 2007). Under these conditions, a non-significant trend of NCAM^−/−^ mice to increasingly approach the unfamiliar interaction partner was detectable. Since olfactory deficits in NCAM^−/−^ mice (Cremer et al., 1994) have been described, we employed an additional social interaction apparatus that allows tactile interaction between test and stranger mouse (i.e., a smaller compartment containing the interaction partner is divided from a larger compartment containing the test animal by a wire mesh fence). Again, NCAM^−/−^ mice displayed comparable levels of interaction to their wild type littermates (data not shown), thus supporting our finding of undisturbed social approach in a neutral test setting, in spite of reported aggression, hyperactivity and olfactory deficits (Cremer et al., 1994; Stork et al., 2000).

Disturbance of attentive stimulus processing is another hallmark of schizophrenia with patients giving and maintaining increased attention to irrelevant stimuli (Weiner, 2003; Lubow, 2005). In rodents, attention deficits can be uncovered in a latent inhibition paradigm, in which repeated stimulus pre-exposure attenuates its association with an unconditioned stimulus (Weiner, 2003). Since NCAM^−/−^ mice display deficits in both auditory cued and contextual fear conditioning (Stork et al., 2000), investigation of latent inhibition in these paradigms could be compromised. However, we could show recently that these deficits can be overcome by a pre-exposure of NCAM^−/−^ mice to a neutral tone (CS–) and the training context (Albrecht et al., 2010). This now allowed us to investigate latent inhibition without confounds through fear memory deficits. Pre-exposure to the CS– as well as to the CS+ resulted in comparable freezing levels between NCAM^−/−^ and NCAM^+/+^ mice. Animals of both genotypes reduced freezing towards the CS+ after CS+ pre-exposure, hence failing to provide evidence for attention deficits in NCAM^−/−^ mice.

Thus, morphological and behavioral data demonstrate that NCAM^−/−^ mice show only deficits in a subset of behavioral domains associated with schizophrenia, while other changes cannot be linked to this disease. Considering different aspects of NCAM function, its regulation through post-translational modifications and its various interaction partners may shed some light on this puzzle.

The extracellular portion of the NCAM molecule (NCAM-EC) can be cleaved by metalloproteases, which results in inhibition of neurite outgrowth and arborization of cortical neurons during development (Brennaman and Maness, 2008). Increased concentrations of NCAM-EC have been also observed in the cerebrospinal fluid of schizophrenic patients (Vawter et al., 2001). Based on this, Pillai-Nair et al. (2005) developed transgenic mice that secrete an extracellular NCAM fragment. These animals display cytomorphological changes in the PFC as well as schizophrenia-related behavioral changes, including hyperactivity, working memory deficits and clozapine-reversible disturbance of prepulse inhibition (PPI). Strikingly, PPI, a pre-attentive sensory gating process, was found unaltered in NCAM^−/−^ mice (Plappert et al., 2005). Thus, mechanisms that control NCAM-mediated cell adhesion through protein expression and shedding appear to differentially affect certain schizophrenia-related phenotypes.

NCAM-mediated adhesion and its functions during development and in certain regions of the mature brain are highly dependent on its posttranslational modification by polysialic acid (PSA). PSA is added to the NCAM core protein through two Golgi-associated polysialyltransferases, ST8SiaII and ST8SiaIV, in a strictly regulated pattern (Bonfanti, 2006). And indeed, single nucleotide polymorphisms in the promoter region of the polysialyltransferases ST8SiaII have been also linked to schizophrenia (Arai et al., 2006; Tao et al., 2007). PSA has been implicated in the NCAM-mediated morphogenic processes in the olfactory bulb and hippocampal mossy fiber system (Hu et al., 1996; Seki and Rutishauser, 1998) as well as the interplay of stress and synaptic plasticity (Bisaz et al., 2009). ST8SiaIV mutant mice showed deficits in hippocampal plasticity (Eckhardt et al., 2000) and spatial memory (Markram et al., 2007), resembling deficits in NCAM^−/−^ mice (Cremer et al., 1994; Stork et al., 2000). This similarity of phenotypes suggests that polysialylated NCAM may be its relevant form in these mnemonic processes.

Interestingly, PSA is able to bind dopamine and a mutated form of ST8SiaII that has been observed also in schizophrenic patients previously, produces a PSA-NCAM type were this function is lost (Isomura et al., 2011). On the other hand, D2 receptor internalization is reduced and D2 surface expression and signaling are increased in NCAM^−/−^ mice (Xiao et al., 2009). Castillo-Gómez et al. (2008) previously found reduced expression of PSA-NCAM after chronic treatment with haloperidol, an antipsychotic drug acting as a dopamine receptor type 2 (D2) antagonist, and an corresponding increase following a D2 agonist 2-(N-Phenethyl-N-propyl) amino-5-hydroxytetralin hydrochloride, In contrast, no D2-dependent expression modulation of the NCAM core protein is observed when PSA is enzymatically removed (Castillo-Gómez et al., 2011). Together these data indicate a differential interaction of the NCAM core protein and NCAM-PSA in the interaction with dopamine receptors, which appears to be negatively correlated with the strength of NCAM-mediated adhesion.

In addition, expression of PSA-NCAM in the PFC is modulated by serotonergic transmission (Varea et al., 2007). We have previously seen that without detectable changes in serotonin levels and 5-HT1A receptor expression, NCAM^−/−^ mice display increased responsiveness to serotonergic anxiolytics (Stork et al., 1999, 2000). This serotonergic hypersensitivity may relate to an interaction of NCAM with inwardly rectifying potassium channels (Kir3.1, Delling et al., 2002), which itself has recently been identified as a schizophrenia susceptibility gene (Yamada et al., 2012). However, the potential role of PSA in these processes is not yet clear.

The compiled evidence from genetic association studies and mutant mouse analysis suggests that the NCAM associated-cell recognition and signaling complex (including FGF receptors: Terwisscha van Scheltinga et al., 2010; and NMDA receptors: Kochlamazashvili et al., 2010) are involved in the development of schizophrenia-related behavioral features. A systematic functional analysis of the different components of this complex and their interaction in nervous system development will help us to better understand the underlying pathophysiology. First steps have been taken by crossbreeding knock out strains for NCAM, ST8SiaII and ST8SiaIV, which revealed that imbalanced polysialylation of NCAM during development causes morphological deficits similar to those in schizophrenic patients (Hildebrandt et al., 2009). The fine differential sensitivity of cognitive functions, neuromodulation and social behavior and to mutations in the NCAM cell recognition complex will certainly help to further dissect these processes.

### Conflict of interest statement

The authors declare that the research was conducted in the absence of any commercial or financial relationships that could be construed as a potential conflict of interest.

## References

[B1] Albrecht A. Bergado-Acosta J. R. Pape H. C. Stork O. (2010). Role of the neural cell adhesion molecule (NCAM) in amygdalo-hippocampal interactions and salience determination of contextual fear memory. Int. J. Neuropsychopharmacol. 13, 661–674 10.1017/S1461145709991106 20003620

[B2] Amann L. C. Gandal M. J. Halene T. B. Ehrlichman R. S. White S. L. McCarren H. S. Siegel S. J. (2010). Mouse behavioral endophenotypes for schizophrenia. Brain Res. Bull. 83, 147–161 10.1016/j.brainresbull.2010.04.008 20433908

[B3] Arai M. Yamada K. Toyota T. Obata N. Haga S. Yoshida Y. Nakamura K. Minabe Y. Ujike H. Sora I. Ikeda K. Mori N. Yoshikawa T. Itokawa M. (2006). Association between polymorphisms in the promoter region of the sialyltransferase 8B (SIAT8B) gene and schizophrenia. Biol. Psychiatry 59, 652–659 10.1016/j.biopsych.2005.08.016 16229822

[B4] Belforte J. E. Zsiros V. Sklar E. R. Jiang Z. Yu G. Li Y. Quinlan E. M. Nakazawa K. (2010). Postnatal NMDA receptor ablation in corticolimbic interneurons confers schizophrenia-like phenotypes. Nat. Neurosci. 13, 76–83 10.1038/nn.2447 19915563 PMC2797836

[B5] Bisaz R. Conboy L. Sandi C. (2009). Learning under stress: a role for the neural cell adhesion molecule NCAM. Neurobiol. Learn. Mem. 91, 333–342 10.1016/j.nlm.2008.11.003 19041949

[B6] Bisaz R. Schachner M. Sandi C. (2011). Causal evidence for the involvement of the neural cell adhesion molecule, NCAM, in chronic stress-induced cognitive impairments. Hippocampus 21, 56–71 10.1002/hipo.20723 19921700

[B7] Bisaz R. Sandi C. (2010). The role of NCAM in auditory fear conditioning and ist modulation by stress: a focus on the amygdala. Genes Brain Behav. 9, 353–364 10.1111/j.1601-183X.2010.00563.x 20059553

[B8] Bisaz R. Sandi C. (2012). Vulnerability of conditional NCAM-deficient mice to develop stress-induced behavioral alterations. Stress 15, 195–206 10.3109/10253890.2011.608226 21939373

[B9] Bonfanti L. (2006). PSA-NCAM in mammalian structural plasticity and neurogenesis. Prog. Neurobiol. 80, 129–164 10.1016/j.pneurobio.2006.08.003 17029752

[B10] Brennaman L. H. Maness P. F. (2008). Developmental regulation of GABAergic interneuron branching and synaptic development in the prefrontal cortex by soluble neural cell adhesion molecule. Mol. Cell. Neurosci. 37, 781–793 10.1016/j.mcn.2008.01.006 18289872 PMC2374759

[B11] Brennaman L. H. Maness P. F. (2010). NCAM in neuropsychiatric and neurodegenerative disorders. Adv. Exp. Med. Biol. 663, 299–317 10.1007/978-1-4419-1170-4_19 20017030

[B12] Bult A. Lynch C. B. (1997). Nesting and fitness: lifetime reproductive success in house mice bidirectionally selected for thermoregulatory nest-building behavior. Behav. Genet. 27, 231–240 9210794 10.1023/a:1025610130282

[B13] Castillo-Gómez E. Gómez-Climent M. A. Varea E. Guirado R. Blasco-Ibáñez J. M. Crespo C. Martínez-Guijarro F. J. Nácher J. (2008). Dopamine acting through D2 receptors modulates the expression of PSA-NCAM, a molecule related to neuronal structural plasticity, in the medial prefrontal cortex of adult rats. Exp. Neurol. 214, 97–111 10.1016/j.expneurol.2008.07.018 18718470

[B14] Castillo-Gómez E. Varea E. Blasco-Ibáñez J. M. Crespo C. Nacher J. (2011). Polysialic Acid is required for dopamine D2 receptor-mediated plasticity involving inhibitory circuits of the rat medial prefrontal cortex. PLoS ONE 6:e29516 10.1371/journal.pone.0029516 22216301 PMC3247286

[B15] Crawley J. N. (2007). Mouse behavioral assays relevant to the symptoms of autism. Brain Pathol. 17, 448–459 10.1111/j.1750-3639.2007.00096.x 17919130 PMC8095652

[B16] Cremer H. Chazal G. Goridis C. Represa A. (1997). NCAM is essential for axonal growth and fasciculation in the hippocampus. Mol. Cell. Neurosci. 8, 323–335 10.1006/mcne.1996.0588 9073395

[B17] Cremer H. Lange R. Christoph A. Plomann M. Vopper G. Roes J. Brown R. Baldwin S. Kraemer P. Scheff S. Barthels D. Rajewsky K. Wille W. (1994). Inactivation of the N-CAM gene in mice results in size reduction of the olfactory bulb and deficits in spatial learning. Nature 367, 455–459 10.1038/367455a0 8107803

[B18] DeLuca J. Donovick P. J. Burright R. G. (1989). Lead exposure, environmental temperature, nesting and consummatory behavior of adult male mice of two ages. Neurotoxicol. Teratol. 11, 7–11 10.1016/0892-0362(89)90079-2 2725444

[B19] Delling M. Wischmeyer E. Dityatev A. Sytnyk V. Veh R. W. Karschin A. Schachner M. (2002). The neural cell adhesion molecule regulates cell-surface delivery of G-protein-activated inwardly rectifying potassium channels via lipid rafts. J. Neurosci. 22, 7154–7164 12177211 10.1523/JNEUROSCI.22-16-07154.2002 PMC6757893

[B20] Eckhardt M. Bukalo O. Chazal G. Wang L. Goridis C. Schachner M. Gerardy-Schahn R. Cremer H. Dityatev A. (2000). Mice deficient in the polysialyltransferase ST8SiaIV/PST-1 allow discrimination of the roles of neural cell adhesion molecule protein and polysialic acid in neural development and synaptic plasticity. J. Neurosci. 20, 5234–5244 10884307 10.1523/JNEUROSCI.20-14-05234.2000 PMC6772332

[B21] Etherton M. R. Blaiss C. A. Powell C. M. Südhof T. C. (2009). Mouse neurexin-1alpha deletion causes correlated electrophysiological and behavioral changes consistent with cognitive impairments. Proc. Natl. Acad. Sci. U.S.A. 106, 17998–18003 10.1073/pnas.0910297106 19822762 PMC2764944

[B22] Fields R. D. Itoh K. (1996). Neural cell adhesion molecules in activity-dependent development and synaptic plasticity. Trends Neurosci. 19, 473–480 10.1016/S0166-2236(96)30013-1 8931273

[B23] Gainetdinov R. R. Mohn A. R. Caron M. G. (2001). Genetic animal models: focus on schizophrenia. Trends Neurosci. 24, 527–533 11506886 10.1016/s0166-2236(00)01886-5

[B24] Gejman P. V. Sanders A. R. Kendler K. S. (2011). Genetics of schizophrenia: new findings and challenges. Annu. Rev. Genomics Hum. Genet. 12, 121–144 10.1146/annurev-genom-082410-101459 21639796

[B25] Gibbons A. S. Thomas E. A. Dean B. (2009). Regional and duration of illness differences in the alteration of NCAM-180 mRNA expression within the cortex of subjects with schizophrenia. Schizophr. Res. 112, 65–71 10.1016/j.schres.2009.04.002 19411161 PMC2722680

[B26] Gray L. J. Dean B. Kronsbein H. C. Robinson P. J. Scarr E. (2010). Region and diagnosis-specific changes in synaptic proteins in schizophrenia and bipolar I disorder. Psychiatry Res. 178, 374–380 10.1016/j.psychres.2008.07.012 20488553

[B27] Hildebrandt H. Mühlenhoff M. Oltmann-Norden I. Röckle I. Burkhardt H. Weinhold B. Gerardy-Schahn R. (2009). Imbalance of neural cell adhesion molecule and polysialyltransferase alleles causes defective brain connectivity. Brain 132, 2831–2838 10.1093/brain/awp117 19443631

[B28] Hu H. Tomasiewicz H. Magnuson T. Rutishauser U. (1996). The role of polysialic acid in migration of olfactory bulb interneuron precursors in the subventricular zone. Neuron 16, 735–743 8607992 10.1016/s0896-6273(00)80094-x

[B29] Isomura R. Kitajima K. Sato C. (2011). Structural and functional impairments of polysialic acid by a mutated polysialyltransferase found in schizophrenia. J. Biol. Chem. 286, 21535–21545 10.1074/jbc.M111.221143 21464126 PMC3122212

[B30] Jaaro-Peled H. Ayhan Y. Pletnikov M. V. Sawa A. (2010). Review of pathological hallmarks of schizophrenia: comparison of genetic models with patients and nongenetic models. Schizophr. Bull. 36, 301–313 10.1093/schbul/sbp133 19903746 PMC2833125

[B30a] Joober R. Boksa P. Benkelfat C. Rouleau G. (2002). Genetics of schizophrenia: from animal models to clinical studies. J. Psychiatry Neurosci. 27, 336–347 12271789 PMC161676

[B31] Kalueff A. V. Keisala T. Minasyan A. Kuuslahti M. Miettinen S. Tuohimaa P. (2006). Behavioural anomalies in mice evoked by “Tokyo” disruption of the Vitamin D receptor gene. Neurosci. Res. 54, 254–260 10.1016/j.neures.2005.12.008 16427152

[B32] Kellendonk C. Simpson E. H. Kandel E. R. (2009). Modeling cognitive endophenotypes of schizophrenia in mice. Trends Neurosci. 32, 347–358 10.1016/j.tins.2009.02.003 19409625 PMC4928481

[B33] Kochlamazashvili G. Senkov O. Grebenyuk S. Robinson C. Xiao M. F. Stummeyer K. Gerardy-Schahn R. Engel A. K. Feig L. Semyanov A. Suppiramaniam V. Schachner M. Dityatev A. (2010). Neural cell adhesion molecule-associated polysialic acid regulates synaptic plasticity and learning by restraining the signaling through GluN2B-containing NMDA receptors. J. Neurosci. 30, 4171–4183 10.1523/JNEUROSCI.5806-09.2010 20237287 PMC5390116

[B34] Kolomeets N. S. Orlovskaya D. D. Uranova N. A. (2007). Decreased numerical density of CA3 hippocampal mossy fiber synapses in schizophrenia. Synapse 61, 615–621 10.1002/syn.20405 17476682

[B35] Laxmi T. R. Stork O. Pape H. C. (2003). Generalisation of conditioned fear and its behavioural expression in mice. Behav. Brain Res. 145, 89–98 10.1016/S0166-4328(03)00101-3 14529808

[B36] Lisman J. (2011). Excitation, inhibition, local oscillations, or large-scale loops: what causes the symptoms of schizophrenia? Curr. Opin. Neurobiol. [Epub ahead of print]. 10.1016/j.conb.2011.10.018 22079494 PMC3302967

[B37] Lu L. Mamiya T. Koseki T. Mouri A. Nabeshima T. (2011). Genetic animal models of schizophrenia related with the hypothesis of abnormal neurodevelopment. Biol. Pharm. Bull. 34, 1358–1363 21881217 10.1248/bpb.34.1358

[B38] Lubow R. E. (2005). Construct validity of the animal latent inhibition model of selective attention deficits in schizophrenia. Schizophr. Bull. 31, 139–153 10.1093/schbul/sbi005 15888432

[B39] Markram K. Gerardy-Schahn R. Sandi C. (2007). Selective learning and memory impairments in mice deficient for polysialylated NCAM in adulthood. Neuroscience 144, 788–796 10.1016/j.neuroscience.2006.10.024 17140740

[B40] Mazzongini R. Zoli M. Tosato S. Lasalvia A. Ruggeri M. (2009). Can the role of genetic factors in schizophrenia be enlightened by studies of candidate gene mutant mice behaviour? World, J. Biol. Psychiatry 10, 778–797 10.1080/15622970902875152 19396727

[B41] Moretti P. Bouwknecht J. A. Teague R. Paylor R. Zoghbi H. Y. (2005). Abnormalities of social interactions and home-cage behavior in a mouse model of Rett syndrome. Hum. Mol. Genet. 14, 205–220 10.1093/hmg/ddi016 15548546

[B42] O'Tuathaigh C. M. Babovic D. O'Meara G. Clifford J. J. Croke D. T. Waddington J. L. (2007). Susceptibility genes for schizophrenia: characterisation of mutant mouse models at the level of phenotypic behaviour. Neurosci. Biobehav. Rev. 31, 60–78 10.1016/j.neubiorev.2006.04.002 16782199

[B43] Pillai-Nair N. Panicker A. K. Rodriguiz R. M. Gilmore K. L. Demyanenko G. P. Huang J. Z. Wetsel W. C. Maness P. F. (2005). Neural cell adhesion molecule-secreting transgenic mice display abnormalities in GABAergic interneurons and alterations in behavior. J. Neurosci. 25, 4659–4671 10.1523/JNEUROSCI.0565-05.2005 15872114 PMC6725026

[B44] Plappert C. F. Schachner M. Pilz P. K. (2005). Neural cell adhesion molecule-null mice are not deficient in prepulse inhibition of the startle response. Neuroreport 16, 1009–1012 15931078 10.1097/00001756-200506210-00025

[B45] Povlsen G. K. Ditlevsen D. K. (2010). The Neural Cell Adhesion Molecule NCAM and Lipid Rafts. Adv. Exp. Med. Biol. 663, 183–198 10.1007/978-1-4419-1170-4_12 20017023

[B46] Robertson G. S. Hori S. E. Powell K. J. (2006). Schizophrenia: an integrative approach to modelling a complex disorder. J. Psychiatry Neurosci. 31, 157–167 16699601 PMC1449879

[B47] Ronn L. C. Berezin V. Bock E. (2000). The neural cell adhesion molecule in synaptic plasticity and ageing. Int. J. Dev. Neurosci. 18, 193–199 10.1016/S0736-5748(99)00088-X 10715574

[B48] Samaco R. C. Fryer J. D. Ren J. Fyffe S. Chao H. T. Sun Y. Greer J. J. Zoghbi H. Y. Neul J. L. (2008). A partial loss of function allele of methyl-CpG-binding protein 2 predicts a human neurodevelopmental syndrome. Hum. Mol. Genet. 17, 1718–1727 10.1093/hmg/ddn062 18321864 PMC2666042

[B49] Seki T. Rutishauser U. (1998). Removal of polysialic acid-neural cell adhesion molecule induces aberrant mossy fiber innervation and ectopic synaptogenesis in the hippocampus. J. Neurosci. 18, 3757–3766 9570806 10.1523/JNEUROSCI.18-10-03757.1998 PMC6793159

[B50] Stork O. Welzl H. Cremer H. Schachner M. (1997). Increased intermale aggression and neuroendocrine response in mice deficient for the neural cell adhesion molecule (NCAM). Eur. J. Neurosci. 9, 1117–1125 9215693 10.1111/j.1460-9568.1997.tb01464.x

[B51] Stork O. Welzl H. Wolfer D. Schuster T. Mantei N. Stork S. Hoyer D. Lipp H. Obata K. Schachner M. (2000). Recovery of emotional behaviour in neural cell adhesion molecule (NCAM) null mutant mice through transgenic expression of NCAM180. Eur. J. Neurosci. 12, 3291–3306 10.1046/j.1460-9568.2000.00197.x 10998113

[B52] Stork O. Welzl H. Wotjak C. T. Hoyer D. Delling M. Cremer H. Schachner M. (1999). Anxiety and increased 5-HT1A receptor response in NCAM null mutant mice. J. Neurobiol. 40, 343–355 10.1002/(SICI)1097-4695(19990905) 10440734

[B53] Sullivan P. F. Keefe R. S. Lange L. A. Lange E. M. Stroup T. S. Lieberman J. Maness P. F. (2007). NCAM1 and neurocognition in schizophrenia. Biol. Psychiatry 61, 902–910 10.1016/j.biopsych.2006.07.036 17161382

[B54] Tamminga C. A. Stan A. D. Wagner A. D. (2010). The hippocampal formation in schizophrenia. Am. J. Psychiatry 167, 1178–1193 10.1038/sc.2008.61 20810471

[B55] Tao R. Li C. Zheng Y. Qin W. Zhang J. Li X. Xu Y. Shi Y. Y. Feng G. He L. (2007). Positive association between SIAT8B and schizophrenia in the Chinese Han population. Schizophr. Res. 90, 108–114 10.1016/j.schres.2006.09.029 17126533

[B56] Terwisscha van Scheltinga A. F. Bakker S. C. Kahn R. S. (2010). Fibroblast growth factors in schizophrenia. Schizophr. Bull. 36, 1157–1166 10.1093/schbul/sbp033 19429845 PMC2963056

[B57] Tordjman S. Drapier D. Bonnot O. Graignic R. Fortes S. Cohen D. Millet B. Laurent C. Roubertoux P. L. (2007). Animal models relevant to schizophrenia and autism: validity and limitations. Behav. Genet. 37, 61–78 10.1007/s10519-006-9120-5 17160702

[B58] Turetsky B. I. Moberg P. J. Yousem D. M. Doty R. L. Arnold S. E. Gur R. E. (2000). Reduced olfactory bulb volume in patients with schizophrenia. Am. J. Psychiatry 157, 828–830 10.1176/appi.ajp.157.5.828 10784482

[B59] Uhlhaas P. J. Haenschel C. Nikoliæ D. Singer W. (2008). The role of oscillations and synchrony in cortical networks and their putative relevance for the pathophysiology of schizophrenia. Schizophr. Bull. 34, 927–943 10.1093/schbul/sbn062 18562344 PMC2632472

[B60] Varea E. Blasco-Ibáñez J. M. Gómez-Climent M. A. Castillo-Gómez E. Crespo C. Martínez-Guijarro F. J. Nácher J. (2007). Chronic fluoxetine treatment increases the expression of PSA-NCAM in the medial prefrontal cortex. Neuropsychopharmacology 32, 803–812 10.1038/sj.npp.1301183 16900104

[B61] Vawter M. P. (2000). Dysregulation of the neural cell adhesion molecule and neuropsychiatric disorders. Eur. J. Pharmacol. 405, 385–395 10.1016/S0014-2999(00)00568-9 11033343

[B62] Vawter M. P. Usen N. Thatcher L. Ladenheim B. Zhang P. VanderPutten D. M. Conant K. Herman M. M. van Kammen D. P. Sedvall G. Garver D. L. Freed W. J. (2001). Characterization of human cleaved N-CAM and association with schizophrenia. Exp. Neurol. 172, 29–46 10.1006/exnr.2001.7790 11681838

[B63] Vicente A. M. Macciardi F. Verga M. Bassett A. S. Honer W. G. Bean G. Kennedy J. L. (1997). NCAM and schizophrenia: genetic studies. Mol. Psychiatry 2, 65–69 9154219 10.1038/sj.mp.4000235 PMC3160977

[B64] Walker E. Mittal V. Tessner K. (2008). Stress and the hypothalamic pituitary adrenal axis in the developmental course of schizophrenia. Annu. Rev. Clin. Psychol. 4, 189–216 Review. 10.1146/annurev.clinpsy.4.022007.141248 18370616

[B65] Weiner I. (2003). The “two-headed” latent inhibition model of schizophrenia: modeling positive and negative symptoms and their treatment. Psychopharmacology (Berl.) 169, 257–297 10.1007/s00213-002-1313-x 12601500

[B66] Xiao M. F. Xu J. C. Tereshchenko Y. Novak D. Schachner M. Kleene R. (2009). Neural cell adhesion molecule modulates dopaminergic signaling and behavior by regulating dopamine D2 receptor internalization. J. Neurosci. 29, 14752–14763 10.1523/JNEUROSCI.4860-09.2009 19940170 PMC6666007

[B67] Yamada K. Iwayama Y. Toyota T. Ohnishi T. Ohba H. Maekawa M. Yoshikawa T. (2012). Association study of the KCNJ3 gene as a susceptibility candidate for schizophrenia in the Chinese population. Hum. Genet. 131, 443–451 10.1007/s00439-011-1089-3 21927946 PMC3277701

